# Progressive Improvement of *T*-Scores in Men with Osteoporosis and Subnormal Serum Testosterone Levels upon Treatment with Testosterone over Six Years

**DOI:** 10.1155/2014/496948

**Published:** 2014-02-13

**Authors:** Ahmad Haider, Ulrich Meergans, Abdulmaged Traish, Farid Saad, Gheorghe Doros, Paul Lips, Louis Gooren

**Affiliations:** ^1^Private Urology Practice, 27570 Bremerhaven, Germany; ^2^Department of Orthopedics, Wesermuende Hospital, 27607 Langen, Germany; ^3^Departments of Biochemistry and of Urology, Boston University School of Medicine, Boston, MA 02118, USA; ^4^Bayer Pharma, Global Medical Affairs Andrology, 13353 Berlin, Germany; ^5^Gulf Medical University School of Medicine, Ajman, UAE; ^6^Department of Biostatistics, Boston University School of Public Health, Boston, MA 02118, USA; ^7^Endocrine Section, Department of Internal Medicine, VU University Medical Center, 1081 HV Amsterdam, The Netherlands; ^8^Chiang Mai 50220, Thailand

## Abstract

Testosterone deficiency leads to bone loss and testosterone treatment has a beneficial effect. This study investigated the effects of normalizing serum testosterone on bone mineral density in 45 men with osteoporosis, diagnosed with testosterone deficiency (serum testosterone levels <12.1 nmol/L, *T*-scores: (mean ± SD) −3.12 ± 0.45, minimum: −4.10, and maximum: −2.60). In a cumulative, prospective, registry study of hypogonadal men (mean age: 53 ± 7 years) they received parenteral testosterone undecanoate of 1000 mg/12 weeks for up to six years. After one year 44 men were included in the registry, after two years 36 men, after three years 32 men, after four years 25 men, after five years 10 men and after six years 4 men. The declining numbers do not reflect drop-out rates but are a result of the registry design. Over the 6 year period there was a significant and progressive improvement of the *T*-scores in these men. Normalizing of serum testosterone leads to an improvement of bone mineral density and this improvement was progressive with the time period of testosterone administration. In this study of 6-years many men with testosterone deficiency suffered from classical diagnoses (Klinefelter's syndrome and testicular pathology) hitherto undiagnosed.

## 1. Introduction

Osteoporosis remains underrecognized and undertreated, more so in men than in women, adding considerably to fracture burden and costs [1].

Though men suffer fewer fractures than women, fracture-related morbidity and mortality are higher in men than in women [2], partly due to greater frailty. Age-related changes in blood androgens and estrogens may contribute to the development or progression of frailty in men [3]. Men usually have higher bone mineral density which contributes to lower fracture incidence in men. Bioavailable androgens and oestrogens regulate these aspects of musculoskeletal sexual dimorphism [4]. Numerous studies point to the significance of normal serum testosterone to maintain bone mineral density (BMD) at various stages of life [5]. Testosterone deficiency leads to loss of BMD and testosterone treatment has a beneficial effect [6]. This study investigated the effects of normalizing serum testosterone on BMD in 45 men with osteoporosis who had consulted an orthopedic surgeon and who were diagnosed as testosterone deficient.

Testosterone deficiency may not be an entity in itself but it may be part of another condition and other constituents of the disease might contribute to bone loss as well. Our patients were suffering from a number of diseases, which, apart from the associated testosterone deficiency, could account for the loss of bone mineral density as well. These conditions included Klinefelter's syndrome, Crohn's disease, alcohol abuse, Hodgkin's lymphoma, kidney transplant, and undescended testis.

## 2. Subjects and Methods

The study was carried out in a Private Urology Practice, Bremerhaven, Germany, between the years 2004 and 2012. Patients had been referred to the Orthopedic Clinic for complaints of the locomotor system. This clinic routinely measures serum testosterone levels in patients with osteoporosis (defined by a *T*-score more than 2.5 standard deviations below the mean value for young adult reference data), especially when patients are young. If, indeed, subnormal serum testosterone levels are encountered, the patient is referred to the Urology Practice for assessment of the etiology of subnormal testosterone levels and possible administration of testosterone, provided there are no contraindications.

The cut-off level for below-normal serum testosterone was determined on the basis of the following considerations: although there is no international consensus as to the normal range of testosterone, clinical data suggest that the normal range of testosterone in adult men is between 12 and 40 nmol/L. A threshold of 12.1 nmol/L was confirmed by an international group of authors based on analyses of several well-known studies in which liquid chromatography tandem mass spectrometry had been used [7].

Men with Klinefelter's syndrome often have reduced bone mass [8, 9]. Remarkably, in one study the loss of bone mineral density in this group did not correlate with serum testosterone or with CAG repeats of the androgen receptor [10]. Muscle strength, previous history of testosterone treatment, age at diagnosis and bone markers were predictors of BMD, but testosterone was not [11]. So, positive effects of testosterone on BMD may be indirect by its well-known effects on muscle.

Men with Crohn's disease show reduced bone mass and bone formation [12]. T cell-mediated increased osteoclast formation from peripheral blood may be a factor [13]. Bone cells from patients with quiescent Crohn's disease show a reduced growth potential and an impeded maturation [14]. In a pilot study we have found a beneficial effect of testosterone on the clinical course of Crohn's disease. The mechanism of this improvement may be immunosuppressive effects of testosterone, reducing chronic inflammation of the intestinal wall in men with Crohn's disease [15].

Men with alcohol abuse may have a bone remodeling imbalance, with a predominant decrease in bone formation. In addition, recent studies have reported new mechanisms by which alcohol may act on bone remodeling, including osteocyte apoptosis, oxidative stress, and Wnt signaling pathway modulation [16].

A single patient had been treated for Hodgkin's lymphoma. Men who have been successfully treated for malignancies earlier in life may develop hypogonadism when they age (>50 year) [17].

Three patients had had a kidney transplant. Impaired renal function maybe associated with loss of BMD and lowered testosterone [18].

Seven patients had a history of undescended testis, most of them bilateral and some with unilateral orchiectomy or testicular atrophy. If not appropriately treated [19], this often leads to a loss of exocrine and endocrine testicular function.

The study was a cumulative, prospective, registry study of men (mean age: 53.07 ± 6.89 years; minimum: 40; maximum: 68 years) with testosterone levels below 12.1 nmol/L. Their *T*-scores were (mean ± SD) −3.12 ± 0.45 (minimum −4.10 and maximum −2.60). They received parenteral testosterone undecanoate of 1000 mg/12 weeks following an initial 6-week interval for up to six years. After six years, 44 men were included in the registry, after five years 36 men, after four years 32 men, after three years 25 men, after two years 10 men, and after one year 4 men. The declining numbers do not reflect drop-out rates but are a result of the registry design. New patients are consecutively entered once they have completed one year of treatment.

Exclusion criteria for testosterone administration included a previous diagnosis of primary or secondary hypogonadism, previous treatment with androgens, bone metastases, prostate cancer, prostate specific antigen (PSA) levels > 4 ng/mL, International Prostate Symptom Score (IPSS) > 19 points, a history of congestive heart failure or recent angina, history of cerebral vascular accident or untreated sleep apnoea.

All initial serum testosterone samples had been obtained between 7.00 and 11.00 h a.m. Serum testosterone levels were measured before testosterone administration, and then before the second injection at 6 weeks, subsequently before the next injection of testosterone undecanoate was due, as a rule; 12 weeks later. Serum testosterone was measured by commercially available chemiluminescent immunoassays.

BMD was measured by using a whole body dual-energy X-ray densitometer (Norland XR-800). All calculations were performed according to the instructions of the manufacturer and standardized procedures. The daily system quality assurance calibration procedures were strictly performed according to the instructions of the manufacturer using a QA Calibration Standard and a QC Spine Phantom. The accuracy of AP Spine Scans and Hip Scans was within 1.0% of industry standard. The *In vivo* Precision of AP Spine Scan is 0.84% (BMD L 2–4 CV). The *In vivo* Precision of Hip Scan is 1.4% (BMD Femoral Neck CV).

Bone mineral density is expressed in g/cm^2^. The individual bone mineral density (BMD) variation was expressed as a *T*-score of measurements of the spine (L2–4) and femoral neck.

Not only was BMD assessed in this study but also the metabolic conditions were followed up. At each visit, body weight, waist circumference, body mass index, serum levels of total cholesterol, HDL, LDL, triglycerides, glucose, and hemoglobin A1c were measured after an overnight fast. Systolic and diastolic blood pressure were measured. C-reactive protein as an indicator of chronic inflammation was determined. The Aging Male Symptoms scale was measured [20] and also the International Index of Erectile Function (erectile function domain) was assessed [21].

A number of safety parameters in relation to testosterone treatment were assessed: prostate volume, serum prostate specific antigen (PSA), residual bladder volume after voiding, the International Prostate Symptoms Score (IPSS), hemoglobin and hematocrit values, and serum alanine aminotransferase (ALT) and aspartate aminotransferase (AST).

Ethical guidelines as formulated by the German “Ärztekammer” (the German Medical Association) for observational studies in patients receiving standard treatment were followed. All subjects consented to be included in the research of their treatment protocol which is in accordance with the Declaration of Helsinki http://www.wma.net. All procedures were carried out with the adequate understanding and written consent of the subjects.

### 2.1. Statistical Analysis

For continuous variables, the mean, median, standard deviation, range, minimum, maximum, and sample size for the overall sample and various groups were reported at each time point. For categorical variables the frequency distribution was reported. We tested the hypotheses regarding change in outcome scores across the study period by fitting a linear mixed effects model to the data. Time (to indicate follow-up interviews) was included as fixed effect in the model. A random effect was included in the model for the intercept. Estimation and test of change in scores were determined by computing the differences in least square means at baseline versus the score at each follow-up interview. Statistical significance was set at *P* < 0.05.

## 3. Results 

Particulars of the 45 patients are presented in Table 1.

Serum testosterone levels rose significantly upon testosterone administration. Trough levels after 1 to 6 years were well above the cutoff for hypogonadal values (12.1 nmol/L), so the values were steady in a eugonadal range (Table 2).

Over the 6-year period there was a significant improvement of the *T*-score in these men (Table 3).

The improvement was progressive: each year of testosterone treatment led to a significant further improvement of the *T*-scores (Table 4) to a state defined as osteopenia (−1 to −2.49 below the mean value for young adult reference data).

**Figure 1:**
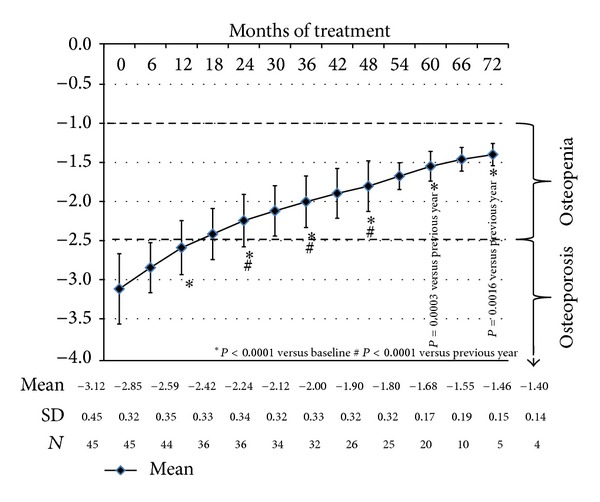
*T*-scores during testosterone administration.

Figure 1 shows that over the 6 year period the *T*-scores of men improved and were no longer classified as osteoporosis but as osteopenia.

Table 4 compares *T*-scores over periods of testosterone treatment from 12 months to 72 months.

Metabolic parameters, blood pressure, and serum CRP showed an improvement over the study period, so did the AMS and IIEF-EF (Table 5).

Safety parameters were assessed (Table 6).

Prostate volume increased slightly but significantly while serum PSA did not change. Values of the IPSS and the residual bladder volume showed a decrease. Hemoglobin and hematocrit rose but remained within normal limits. Serum ALT and AST decreased significantly.

## 4. Discussion

In this study men with osteoporosis and lower-than-normal serum testosterone were treated with testosterone undecanoate whereupon serum testosterone levels normalized. Their copathologies varied strongly but testosterone deficiency was a common denominator. Other elements of their disease may have contributed to their bone loss as well. But in all men, an improvement of *T*-scores was found upon testosterone treatment with a significant progression over duration of the testosterone treatment. In fact, while all men had been in the category of osteoporosis at baseline, the mean *T*-scores improved to a level which is classified as osteopenia. Part of the positive effects may have been due to the positive effects of testosterone on muscle [22–24]. The increase of *T*-scores was impressive, amounting to 1.5 points. The calculated fracture risk reduction would be at least 50%. Several studies have documented the beneficial effects of testosterone administration on BMD in hypogonadal men [9, 25–31]. One study demonstrated that adequacy of testosterone is pivotal for the restoration of BMD in men [32]. Serum testosterone after administration of testosterone undecanoate was well in the eugonadal range. It is now well documented in the literature that a chronic state of testosterone deficiency leads to a host of pathologies in (aging) men [33, 34]. These pathologies (metabolic syndrome, inflammatory factors, lower urinary tract symptoms, erectile dysfunction, and psychological functions were also assessed in this study and showed improvements over the duration of the study (Table 5).

A significant part of the group studied were relatively young men, particularly the men with Klinefelter's syndrome. In a recent study it was reported that over one-third of men less than 50 years with testosterone deficiency and infertility or sexual dysfunction were found to have reduced BMD. These were no men with a classical condition of testosterone deficiency (Klinefelter's or Kallmanns syndrome etcs). Over a mean follow-up of 2.5 years testosterone therapy in this population increased BMD [35]. These findings argue for measurement of BMD in relatively young hypogonadal men.

We noted not only an improvement of *T*-scores in the men studied but also a progressive improvement of the metabolic status of the study subjects: body weight, BMI and waist circumference improved progressively which was also the case for lipids, blood pressure, hemoglobin A1c, and a parameter of inflammation. These improvements upon testosterone treatment have been reported from our clinic [36–38]. There was a progressive improvement of serum ALT and AST probably indicating improvement of liver steatosis, as we have reported earlier [39]. Sexual function, as measured by International Index of Erectile Function, improved significantly over time as reported earlier [40, 41]. We noted also an improvement of the Aging Male Symptoms scale, also reported in other studies [31]. Earlier we have reported that there is a relation with parameters of inflammation such C-reactive protein [37].

Serious attention was paid to safety aspects of testosterone administration to men, for a part elderly, in this study. No malignancies occurred in the study population. We noted a slight increase in prostate volume over the study period, which also occurs in men not treated with testosterone, simply because they age [42, 43]. Serum PSA did not change significantly over the study period. Remarkably, residual volume in the bladder and scores of International Prostate Symptom Score improved considerably upon testosterone treatment, a positive effect earlier reported from this clinic [44]. As expected, hemoglobin levels and the hematocrit rose upon testosterone treatment but remained within safe limits as reported earlier [45]. There was no indication of a disturbance of liver function. The safety of testosterone administration to elderly men is now well documented in the literature [46].

One of the limitations of this study is the nature of the registry design. This single-center, open-label study is not a randomized controlled study and therefore limits the scope of interpretation of the presented findings. Simply, subjects were treated in a urology clinical setting. It is an observational study, not blinded, and not placebo-controlled. The study was not primarily designed to monitor the effects of normalizing serum testosterone in hypogonadal men on bone mineral density and was performed in a urology setting. We never expected to have such a large number of patients with osteoporosis, and, therefore, biomarkers of bone remodeling were not measured.

## 5. Conclusions

This study analyzed testosterone deficiency in a population of middle-aged to elderly men who were referred to an orthopedic clinic with complaints of the locomotor system and were diagnosed with osteoporosis. Their copathologies varied widely but a state of testosterone deficiency was a common denominator. In spite of the varieties of etiologies of their testosterone deficiency, they all benefited from testosterone treatment restoring their serum testosterone to the mid normal range of reference values. Osteoporosis improved to osteopenia. It would appear from our study that men attending an orthopedic clinic should be assessed for testosterone deficiency, first on clinical grounds whether they have copathology associated with testosterone deficiency, and second by confirmation of measurement of testosterone. In case of lower-than-normal serum testosterone, treatment with testosterone not only improved their bone mineral density but benefited also their metabolic state, mood and sexual functioning. Risks of testosterone administration to elderly men are acceptable and manageable.

## Figures and Tables

**Table 1:** Patient characteristics and gains in *T*-score upon testosterone treatment.

No	Year of birth	Diagnosis	Initial testosterone nmol/L	Initial *T*-score	Months of testosterone	*T*-score
1	1950	Alcohol abuse	11.1	−3.1	39	−2.1
2	1949	Alcohol abuse	12.1	−3.1	57	−2.1
3	1951	Alcohol abuse	11.8	−3.9	48	−3.1
4	1964	Alcohol abuse	7.6	−3.9	39	−1.9
5	1955	Alcohol abuse	8.7	−2.9	27	−2.1
6	1948	Alcohol abuse	8.3	−2.7	48	−1.5
7	1962	Klinefelter's syndrome	9.7	−2.9	75	−1.3
8	1952	Klinefelter's syndrome	7.6	−3.7	75	−1.5
9	1969	Klinefelter's syndrome	8.3	−3.7	15	−1.9
10	1967	Klinefelter's syndrome	10.1	−3.8	12	−2.1
11	1961	Klinefelter's syndrome	10.1	−3.4	12	−2.9
12	1959	Klinefelter's syndrome	10.7	−3.6	15	−2.1
13	1963	Klinefelter's syndrome	9.4	−3.8	15	−1.9
14	1963	Klinefelter's syndrome	9.0	−4.1	15	−2.1
15	1959	Klinefelter's syndrome	7.3	−3.6	12	−2.9
16	1967	Klinefelter's syndrome	8.3	−3.7	12	−2.5
17	1971	Klinefelter's syndrome	10.7	−3.1	9	−2.7
18	1952	Klinefelter's syndrome	10.1	−2.8	57	−1.8
19	1955	Klinefelter's syndrome	11.1	−2.6	54	−1.6
20	1959	Klinefelter's syndrome	8.0	−2.9	54	−1.7
21	1955	Klinefelter's syndrome	10.7	−2.9	51	−1.7
22	1961	Klinefelter's syndrome	11.1	−3.1	45	−1.8
23	1957	Klinefelter's syndrome	8.3	−2.8	57	−1.7
24	1965	Klinefelter's syndrome	11.1	−2.8	36	−1.7
25	1950	Klinefelter's syndrome	8.7	−2.7	63	−1.5
26	1965	Klinefelter's syndrome	10.7	−2.9	39	−1.9
27	1965	Klinefelter's syndrome	11.4	−2.8	39	−1.8
28	1951	Klinefelter's syndrome	10.1	−2.8	51	−1.5
29	1949	Crohn's disease	7.3	−2.9	54	−1.8
30	1949	Crohn's disease	7.3	−2.9	51	−1.8
31	1947	Crohn's disease	6.6	−2.7	63	−1.9
32	1950	Crohn's disease	10.7	−2.9	57	−1.4
33	1959	Crohn's disease/Klinefelter's syndrome	7.3	−2.9	33	−1.8
34	1946	Primary hypogonadism	8.3	−2.6	60	−1.6
35	1962	Primary hypogonadism	9.7	−2.7	30	−1.7
36	1951	Primary hypogonadism	10.1	−2.8	54	−1.7
37	1949	Primary hypogonadism	7.6	−2.6	60	−1.5
38	1950	Primary hypogonadism	7.3	−2.8	57	−1.7
39	1960	Primary hypogonadism	7.3	−3.7	36	−1.8
40	1938	Primary hypogonadism	8.7	−3.7	75	−1.5
41	1952	Primary hypogonadism	11.1	−2.9	24	−1.8
42	1950	Primary hypogonadism	12.1	−2.7	60	−1.3
43	1939	Primary hypogonadism	11.8	−2.7	57	−1.5
44	1941	Renal insufficiency	7.6	−2.9	69	−1.2
45	1949	Renal insufficiency	8.7	−3.8	72	−1.5

**Table 2:** Serum levels of testosterone over 72 months of testosterone treatment.

Visit month testosterone	*N*	Mean ± SD (nmol/L)	Minimum	Maximum
0 months	45	9.35 ± 1.5	6.59	12.13
12 months	44	17.3 ± 2.66	13.52	23.92
24 months	36	17.23 ± 2.22	14.21	23.58
36 months	32	16.83 ± 1.91	13.52	20.46
48 months	25	17.28 ± 2.09	14.56	21.84
60 months	9	16.95 ± 1.87	14.21	19.76
72 months	4	14.91 ± 0.63	14.21	15.60

**Table 3:** *T*-scores over 72 months of testosterone treatment.

Visit month—*T*-Scores	*N*	Mean ± SD	Minimum	Maximum
0 month	45	−3.12 ± 0.45	−4.10	−2.60
12 months	44	−2.59 ± 0.35	−3.80	−2.10
24 months	36	−2.24 ± 0.34	−3.70	−1.80
36 months	32	−2 ± 0.32	−3.50	−1.70
48 months	25	−1.8 ± 0.32	−3.10	−1.50
60 months	10	−1.55 ± 0.19	−1.90	−1.30
72 months	4	−1.4 ± 0.14	−1.50	−1.20

**Table 4:** Comparison of *T*-scores over periods of testosterone treatment.

Comparison	Difference ± SE	*P*-Value
12 Months—Baseline	0.53 ± 0.04	<0.0001
24 Months—Baseline	0.85 ± 0.04	<0.0001
36 Months—Baseline	1.13 ± 0.04	<0.0001
48 Months—Baseline	1.34 ± 0.04	<0.0001
60 Months—Baseline	1.58 ± 0.06	<0.0001
24 Months—12 Months	0.32 ± 0.04	<0.0001
36 Months—24 Months	0.28 ± 0.04	<0.0001
48 Months—36 Months	0.21 ± 0.05	<0.0001
60 Months—48 Months	0.24 ± 0.07	0.0003
72 Months—60 Months	0.33 ± 0.1	0.0016

**Table 5:** Effects of testosterone on metabolic variables (means ± SD).

	Visit month							Difference 60
	0 month	12 months	24 months	36 months	48 months	60 months	72 months	months-baseline
*N*	45	44	36	32	25	10	4	
WC (cm)	101.6 ± 6.4	100.1 ± 5.5	99.2 ± 4.9	98.4 ± 4.2	97.7 ± 4.3	97.7 ± 5.2	95.8 ± 4.4	−6.3 ± 0.5^1^
Weight (kg)	97.1 ± 14.4	94.1 ± 13.3	92.2 ± 11.9	91.0 ± 10.7	86.6 ± 9.8	87.0 ± 10.2	86.8 ± 7.9	−14.1 ± 1.1^1^
BMI	29.9 ± 5.0	29.0 ± 4.7	28.7 ± 4.2	28.3 ± 3.8	27.0 ± 3.4	27.5 ± 4.1	27.7 ± 2.5	−4.4 ± 0.3^1^
Fasting glucose (mg/dL)	5.47 ± 0.36	5.32 ± 0.36	5.35 ± 0.51	5.33 ± 0.32	5.34 ± 0.18	5.39 ± 0.13	5.36 ± 0.06	−0.15 ± 0.11^2^
Total cholesterol (mg/dL)	248 ± 29	195 ± 21	185 ± 17	185 ± 14	183 ± 14	187 ± 12	193 ± 10	−63 ± 5^1^
HDL (mg/dL)	50 ± 15	55 ± 15	58 ± 14	59 ± 15	57 ± 14	57 ± 17	60 ± 19	8 ± 1^1^
LDL (mg/dL)	159 ± 32	143 ± 29	131 ± 25	133 ± 24	132 ± 20	125 ± 28	122 ± 33	−30 ± 3^1^
Triglycerides (mg/dL)	241 ± 32	195 ± 25	186 ± 18	184 ± 13	190 ± 14	188 ± 14	190 ± 10	−55 ± 5^1^
Systolic BP (mm Hg)	136 ± 10	128 ± 9	126 ± 7	127 ± 7	128 ± 5	127 ± 3	126 ± 5	−12 ± 2^1^
Diastolic BP (mm Hg)	83 ± 7	76 ± 5	74 ± 4	75 ± 4	74 ± 4	75 ± 3	71 ± 4	−9 ± 1^1^
AMS	47 ± 10	21 ± 3	18 ± 1	17 ± 1	17 ± 1	17 ± 1	18 ± 1	−30 ± 1^1^
IIEF-EF	19.5 ± 5.1	23.0 ± 4.6	23.8 ± 5.1	23.8 ± 4.7	24.2 ± 4.5	26.6 ± 1.8	28.5 ± 0.6	4.8 ± 0.6^1^
*N*	19	7	7	6	3	3	2	
HbA1c (%)	5.9 ± 1.3	6.5 ± 1.4	6.2 ± 1.0	5.8 ± 1.0	6.4 ± 0.5	6.1 ± 0.6	6.4 ± 0.8	1.4 ± 0.2^1^
*N*	45	19	15	17	12	5	1	
CRP (mg/L)	6.2 ± 7.6	3.7 ± 5.4	2.8 ± 3.7	4.2 ± 4.4	2.8 ± 2.9	1.7 ± 1.5	2.2	−7.7 ± 1^1^

^1^ *P* < 0.0001.

^2^Non significant.

WC: Waist Circumference.

BMI: Body Mass Index.

HDL: High Density Lipoprotein.

LDL: Low Density Lipoprotein.

BP: Blood Pressure.

AMS: Aging Male Symptoms Scale.

IIEF-EF: International Index of Erectile Function.

HbA1c: Hemoglobin A1c.

CRP: C-reactive protein.

**Table 6:** Safety parameters of men receiving testosterone treatment (means ± SD).

Visit month	*N*	Prostate volume (mL)	*N*	PSA (ng/dL)	*N*	Residual volume (mL)	*N*	IPSS score	*N*	Hemoglobin (g/L)	*N*	Hematocrit (%)	*N*	ALT (U/L)	*N*	AST (U/L)
0 month	45	19.8 ± 7.4	45	0.96 ± 1.05	41	26.0 ± 17.0	45	3.1 ± 3.0	45	14.6 ± 0.6	45	44.4 ± 2.0	45	44.8 ± 21.7	45	43.7 ± 20.9
12 months	16	21.9 ± 9.8	20	1.26 ± 1.00	39	18.7 ± 14.2	43	2.1 ± 2.1	44	14.9 ± 0.5	44	46.4 ± 2.2	44	29.6 ± 14.2	44	27.7 ± 12.6
24 months	10	26.8 ± 10.6	17	1.45 ± 1.05	33	15.5 ± 10.6	35	1.9 ± 1.8	36	15.0 ± 0.4	36	47.7 ± 1.7	36	26.6 ± 11.9	36	26.8 ± 9.6
36 months	12	26.9 ± 9.2	15	1.40 ± 1.12	29	14.7 ± 9.0	32	1.8 ± 1.4	32	15.1 ± 0.4	32	48.4 ± 1.8	32	24.6 ± 8.4	32	25.6 ± 7.9
48 months	8	29.3 ± 10.6	9	1.37 ± 1.19	22	13.0 ± 5.5	25	1.4 ± 0.9	25	15.1 ± 0.4	25	48.4 ± 1.4	25	25.2 ± 5.6	25	25.4 ± 5.9
60 months	3	27.7 ± 16.2	4	1.59 ± 1.20	10	13.5 ± 6.7	10	1.8 ± 1.1	10	15.1 ± 0.4	10	48.0 ± 1.8	10	22.8 ± 5.2	10	22.4 ± 4.8
72 months	3	24.3 ± 16.7	3	1.04 ± 1.21	4	12.5 ± 5.0	4	1.8 ± 1.0	4	15.2 ± 0.3	4	49.0 ± 1.8	4	22.0 ± 3.5	4	21.0 ± 2.9
Difference 60 months-baseline		3.1 ± 0.5^1^		0.15 ± 0.09^2^		−19.12 ± 2.23^1^		−2.5 ± 0.4^1^		0.54 ± 0.1^1^		3.8 ± 0.4^1^		−28.4 ± 2.5^1^		−25.5 ± 2.3^1^

^1^ *P* < 0.0001.

^2^Non significant.

PSA: Prostate specific antigen.

IPSS: International prostate symptom score.

ALT: Alanine aminotransferase.

AST: Aspartate aminotransferase.
